# Hexosamine pathway activation improves memory but does not extend lifespan in mice

**DOI:** 10.1111/acel.13711

**Published:** 2022-09-19

**Authors:** Kira Allmeroth, Matías D. Hartman, Martin Purrio, Andrea Mesaros, Martin S. Denzel

**Affiliations:** ^1^ Max Planck Institute for Biology of Ageing Cologne Germany; ^2^ CECAD ‐ Cluster of Excellence University of Cologne Cologne Germany; ^3^ Center for Molecular Medicine Cologne University of Cologne Cologne Germany; ^4^ Altos Labs, Cambridge Institute of Science Granta Park, Great Abington Cambridge UK

**Keywords:** GFAT1, hexosamine biosynthetic pathway, memory, metabolism, mouse survival

## Abstract

Glucosamine feeding and genetic activation of the hexosamine biosynthetic pathway (HBP) have been linked to improved protein quality control and lifespan extension. However, as an energy sensor, the HBP has been implicated in tumor progression and diabetes. Given these opposing outcomes, it is imperative to explore the long‐term effects of chronic HBP activation in mammals. Thus, we asked if HBP activation affects metabolism, coordination, memory, and survival in mice. N‐acetyl‐D‐glucosamine (GlcNAc) supplementation in the drinking water had no adverse effect on weight in males but increased weight in young females. Glucose or insulin tolerance was not affected up to 20 months of age. Of note, we observed improved memory in young male mice supplemented with GlcNAc. Survival was not changed by GlcNAc treatment. To assess the effects of genetic HBP activation, we overexpressed the pathway's key enzyme GFAT1 and a constitutively activated mutant form in all mouse tissues. We detected elevated levels of the HBP product UDP‐GlcNAc in mouse brains, but did not find any effects on behavior, memory, or survival. Together, while dietary GlcNAc supplementation did not extend survival in mice, it positively affected memory and is generally well tolerated.

## Graphical abstract

The activation of the hexosamine biosynthetic pathway (HBP) is linked to improved protein quality control and lifespan extension. However, as an energy sensor, the HBP has been implicated in tumor progression and diabetes. Using chronic HBP activation and lifelong observation, we find no effect on survival but enhanced memory function in young male mice supplemented with GlcNAc.

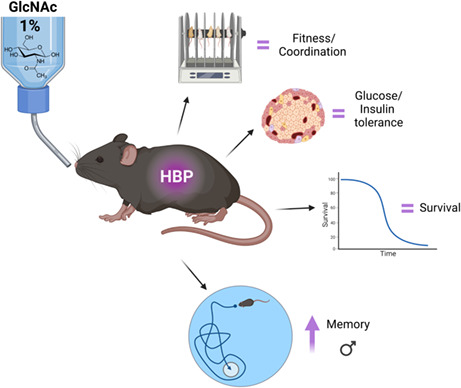

## 1 INTRODUCTION

**FIGURE 1:**
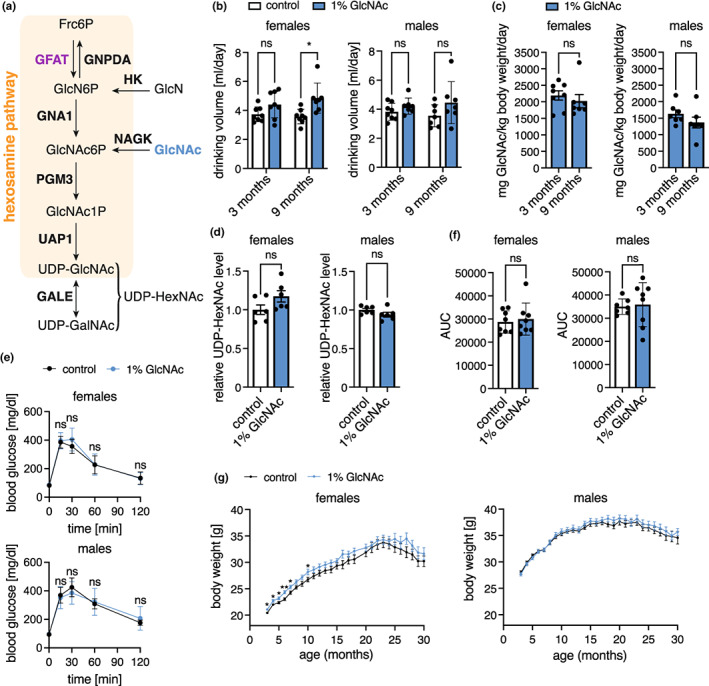
Hexosamine biosynthetic pathway activation by GlcNAc feeding does not have adverse effects in mice. (a) Schematic representation of the hexosamine biosynthetic pathway. The rate‐limiting enzyme glutamine fructose‐6‐phosphate amidotransferase (GFAT) is depicted in purple, GlcNAc is marked in blue. GNPDA: Glucosamine‐6‐phosphate isomerase 1; GNA1: Glucosamine‐6‐phosphate N‐acetyltransferase; PGM3: Phosphoacetylglucosamine mutase; UAP1: UDP‐N‐acetylhexosamine pyrophosphorylase; GALE: UDP‐glucose 4‐epimerase; HK: Hexokinase; NAGK: N‐acetyl‐D‐glucosamine kinase. (b) Drinking volume of control (white) and GlcNAc‐treated mice (blue) of both sexes at 3 and 9 months of age. Data are presented as mean ± SD (*n* ≥ 7). Two‐way ANOVA, Tukey's post‐test; *p < 0.05; ns: Not significant. (c) GlcNAc consumption in mg/kg body weight per day of mice of both sexes at 3 and 9 months of age. Data are presented as mean ± SD (*n* ≥ 7). Unpaired t‐test; ns: Not significant. (d) Relative UDP‐HexNAc levels in hemibrains of control (white) and GlcNAc‐treated mice (blue) of both sexes. Data are presented as mean ± SEM (*n* = 6). Unpaired t‐test; ns: Not significant. (e) Blood glucose concentration at 0 (fasting), 15, 30, 60, and 120 min after intraperitoneal injection of glucose solution (2 g/kg body weight) of control (black) and GlcNAc‐treated mice (blue) of both sexes at 10 months of age. Data are presented as mean ± SD (*n* ≥ 7). Multiple unpaired t‐tests; ns: Not significant (f) area under the curve (AUC) calculated using data shown in (e). Data are presented as mean ± SD (*n* ≥ 7). Unpaired t‐test; ns: Not significant (g) Body weight of control (black) and GlcNAc‐treated mice (blue) of both sexes from 3 to 30 months of age. The data point at 19 months (females) is missing since the body weight measurements were not performed for this time point. Data are presented as mean ± SD (*n* ≥ 13). Multiple unpaired t‐tests; **p < 0.01; *p < 0.05; only significant changes are indicated

The hexosamine biosynthetic pathway (HBP) is an anabolic pathway that consumes fructose‐6‐phosphate (Fru6P), glutamine, acetyl‐CoA, and UTP to produce the high energy molecule uridine 5′‐diphospho‐N‐acetyl‐D‐glucosamine (UDP‐GlcNAc) (Figure 1a). The HBP thus requires carbohydrate, aminoacidic, lipidic, and nucleotide donors and is centrally positioned at a cross‐roads of energy metabolism (Wells et al., 2003). The first HBP step is catalyzed by glutamine fructose‐6‐phosphate amidotransferase (GFAT), that employs L‐glutamine (Gln) as a nitrogen donor to convert Fru6P to D‐glucosamine‐6‐phosphate (GlcN6P), diverting 2%–3% of cellular glucose to the HBP (Marshall et al., 1991). The HBP products UDP‐GlcNAc and its epimer UDP‐GalNAc are precursors for biopolymer synthesis and protein glycosylation (Hanisch, 2001; Hart, 1997; Parodi, 2000).

In eukaryotes, GFAT1 is inhibited by UDP‐GlcNAc in a metabolic feedback loop. We delineated several single amino acid substitutions in GFAT1 that increase activity through loss of UDP‐GlcNAc inhibition, resulting in elevated HBP flux and UDP‐GlcNAc accumulation (Denzel et al., 2014; Horn et al., 2020; Ruegenberg et al., 2020, 2021). Two alternative reactions feed into the HBP, bypassing the finely regulated GFAT‐mediated first step: glucosamine (GlcN) can be incorporated through its phosphorylation via hexokinase to generate GlcN6P (Stocchi et al., 1981), and N‐acetyl‐D‐glucosamine (GlcNAc) can be incorporated through its phosphorylation via N‐acetyl‐D‐glucosamine kinase producing GlcNAc6P (Weihofen et al., 2006). Overall, HBP activation can be achieved through GFAT1 overexpression or through metabolite supplementation.

O‐GlcNAc modification links UDP‐GlcNAc availability, hence HBP activity, to protein function through post‐translational modifications. Hyper‐O‐GlcNAcylation is observed in many cancer types, suggesting that it is a key molecular event in tumor formation, progression, aggressiveness, and potentially a cancer hallmark (Fardini et al., 2013). Elevated GFAT1 has been associated with poor overall survival in patients suffering hepatocellular carcinoma (Li et al., 2017) and, strikingly, GFAT inhibitors like the diazoderivative of serine, azaserine, and 6‐diazo‐5‐oxo‐L‐norleucine (DON) decrease HBP flux and exhibit anti‐tumor activity (Lemberg et al., 2018). The HBP has also been shown to be involved in diabetes; HBP activation in young rats by GlcN infusion leads to insulin resistance and enhanced transcription factor glycosylation, resembling an aging phenotype (Einstein et al., 2008; Marshall et al., 1991). Moreover, GlcN impairs the GLUT4 glucose transporter (whose expression is stimulated by insulin), resembling an insulin‐resistant state (Baron et al., 1995). These data highlight the importance of monitoring potential adverse side effects upon chronic HBP activation. Previous observations, however, also support a plausible role of the HBP in longevity and prevention of age‐related pathologies (Denzel & Antebi, 2015). The HBP is involved in protein quality control and its activation leads to improved protein homeostasis and lifespan extension in *Caenorhabditis elegans*: worms with GFAT‐1 gain‐of‐function (gof) mutations have increased UDP‐GlcNAc levels and are long‐lived (Denzel et al., 2014). Strikingly, this lifespan extension is recapitulated by GlcNAc feeding in a dose‐dependent manner (Denzel et al., 2014). Elevating HBP metabolite levels enhances ER protein quality control, ER‐associated protein degradation (ERAD), and autophagy (Denzel et al., 2014; Shintani et al., 2018). Of note, worms overexpressing WT GFAT‐1 or supplemented with GlcNAc activate the integrated stress response (ISR) (Horn et al., 2020). In mammalian cells, mild HBP activation likewise induces the ISR: the PERK branch is activated leading to increased phosphorylation of the α subunit of eukaryotic initiation factor 2 (eIF2α) and upregulation of the transcription factor ATF4 (Horn et al., 2020). In cells, HBP activation has a protective role against proteotoxicity in a PERK‐ and autophagy‐dependent manner and muscle cell‐specific HBP activation rescues polyQ‐mediated toxicity in worms, a process that depends on ATF4 (Horn et al., 2020). GlcN supplementation likewise induces ER stress in rat cells (Lombardi et al., 2012), and it leads to eIF2α phosphorylation through the PERK branch with a concomitant mRNA translation arrest (Kline et al., 2006). Overall, these data suggest a protective role of HBP activation in various species. Thus, there is considerable interest in the potential health benefits of dietary supplementation with GlcN or other HBP metabolites in people. However, the effects of chronic HBP activation in mice remain unknown.

Here, we tested the long‐term effects of GlcNAc supplementation and genetic HBP activation in mice. Based on our results in *C. elegans*, we hypothesized that the elevation of HBP flux extends mouse lifespan. To assess the physiological effects of HBP activation, we examined markers of health, glucose metabolism, general behavior, and memory. HBP activation had no adverse effects in mice. Importantly, the interventions did not interfere with glucose metabolism. Mice supplemented with dietary GlcNAc or overexpressing GFAT1 did not exhibit lifespan extension. Nevertheless, we observed memory improvement in young male mice fed with GlcNAc, suggesting a beneficial effect of HBP activation in mice.

## 2 RESULTS

### 2.1 HBP pathway activation by GlcNAc feeding does not have adverse effects in mice

To chronically activate the HBP, we supplemented the drinking water of C57BL/6J mice with 1% GlcNAc starting at 8 weeks of age. Females consumed more GlcNAc‐supplemented water compared with controls at 9 months of age; males, however, did not show any difference in water consumption (Figure 1b). The drinking volume of around 4 ml per day corresponds to a GlcNAc consumption of 1500‐2000 mg*kg^−1^ body weight per day (Figure 1c). GlcNAc supplementation in the drinking water had no effect on food intake (Figure S1a). We used LC–MS to quantify UDP‐HexNAc levels (combined UDP‐GlcNAc and UDP‐GalNAc) in brain lysates from 9 months old mice to test effects of dietary supplementation. Surprisingly, after 7 months of GlcNAc feeding, the UDP‐HexNAc levels were not affected in mouse brains (Figure 1d); similarly, liver samples did not show increased levels of UDP‐HexNAc (data not shown).

Because elevated flux through the HBP has been associated with insulin resistance and diabetic complications in rats (Han et al., 2003; Rossetti et al., 1995), we tested the effect of chronic GlcNAc supplementation on glucose utilization by measuring blood glucose clearance and insulin‐stimulated glucose utilization. Chronic GlcNAc intake had no detectable effect on either blood glucose clearance or insulin response in 10 months old mice (Figure 1e,f and Figure S1c), or 20 months old mice (Figure S1b,c). In 4 months old females, however, we observed slightly higher blood glucose levels 30 and 60 min after the injection of insulin (Figure‐S1c). Nevertheless, overall, we conclude that GlcNAc supplementation did not alter insulin signaling.

We further evaluated the impact of GlcNAc supplementation on body weight for 30 months. While males did not display any difference in body weight, GlcNAc supplemented females showed increased body weight compared with controls up to 10 months of age (Figure 1g). Indirect calorimetric measurements revealed an increased respiratory exchange ratio (RER) in 9 months old female mice during the day, while no difference was detected in age‐matched male mice (Figure S1d). This increased RER, as well as the effects on body weight, might be caused by the elevated GlcNAc consumption (Figure 1b,c), which was also sex‐specific. Despite these effects, female mice did not display changes in insulin signaling after 4 months of age. Therefore, overall, our data suggest that chronic GlcNAc supplementation does not have negative side effects in mice.

### 2.2 GlcNAc supplementation does not affect coordination or neuromuscular function

**FIGURE 2:**
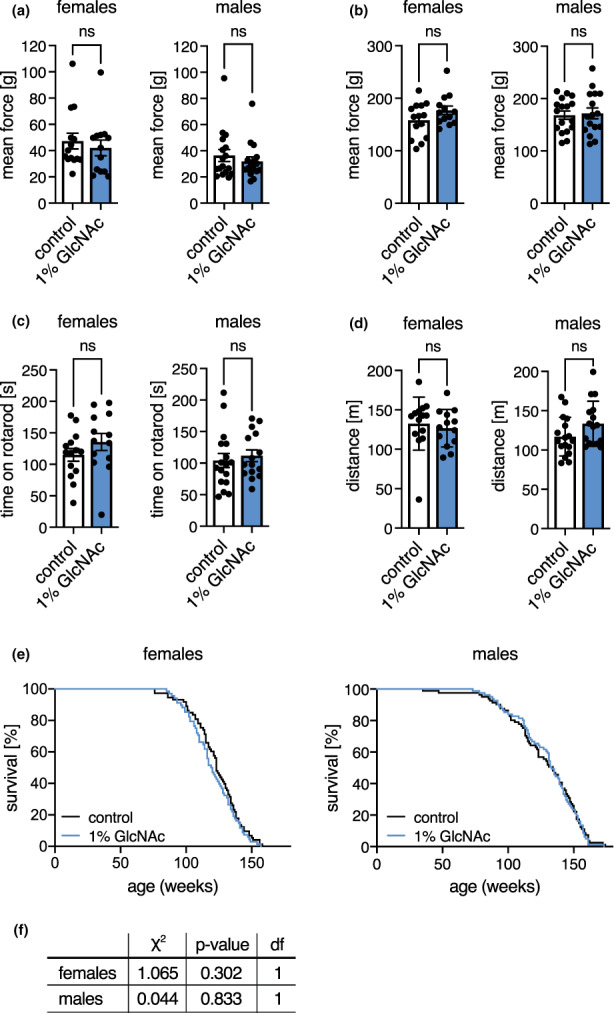
GlcNAc supplementation does not influence the fitness of mice. (a) Mean force measured in a grip strength test with two paws of control (white) and GlcNAc‐treated mice (blue) of both sexes at 6 months of age. (b) Mean force measured in a grip strength test with four paws of control (white) and GlcNAc‐treated mice (blue) of both sexes at 6 months of age. (c) Maximal time on a rotarod of control (white) and GlcNAc‐treated mice (blue) of both sexes at 6 months of age. (a‐c) Data are presented as mean ± SEM (*n* ≥ 13). (d) Maximal distance on a treadmill of control (white) and GlcNAc‐treated mice (blue) of both sexes from at 6 months of age. Data are presented as mean ± SD (*n* ≥ 13). (a‐d) Unpaired t‐test; ns: Not significant (e) Lifespan analysis of control (black) and GlcNAc‐treated mice (blue) of both sexes (females: *N* = 69; males: *N* = 82). (f) Cumulative incidence calculated by Gray's test based on the lifespan analysis shown in (e). Df: Degrees of freedom

Having excluded negative effects of dietary GlcNAc supplementation on the metabolic health of mice, we next aimed to test possible effects of GlcNAc supplementation on coordination and basic neuromuscular function. To this end, the fitness of mice at 6 months of age was analyzed using the rotarod, and by assessing grip strength and treadmill endurance. Of note, GlcNAc supplementation did not affect grip strength (Figure 2a,b), rotarod performance (Figure 2c), or forced maximal endurance on a treadmill (Figure 2d). Thus, fitness and locomotion of mice were not changed by GlcNAc supplementation.

As GlcNAc supplementation extends *C. elegans* lifespan (Denzel et al., 2014) and showed no adverse effects in mice, we supplemented mice of both sexes with GlcNAc in the drinking water from week 8 until they died to assess a possible modulation of murine lifespan. Surprisingly, GlcNAc feeding did not affect mouse survival (Figure 2e,f). In sum, we conclude that chronic HBP activation by GlcNAc feeding does not have adverse side effects on general fitness and health in mice.

### 2.3 GlcNAc feeding improves memory of young male mice

**FIGURE 3:**
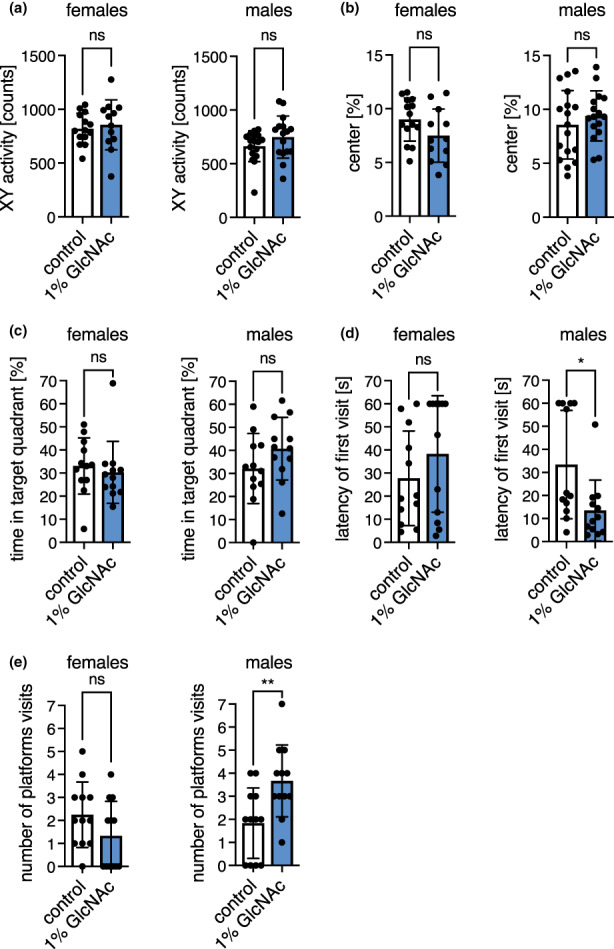
GlcNAc feeding improves memory of young male mice. (a) XY activity measured in the open field test of control (white) and GlcNAc‐treated mice (blue) of both sexes at 6 months of age. (b) Percent of distance spent in the center of the open field of control (white) and GlcNAc‐treated mice (blue) of both sexes at 6 months of age. (a‐b) Data are presented as mean ± SD (*n* ≥ 12). (c) Percent of time spend in the target quadrant, (d) Latency of the first platform visit, and (e) Number of platform visits upon removal of the hidden platform in the Morris water maze test of control (white) and GlcNAc‐treated mice (blue) of both sexes at 4 months of age. (c‐e) Data are presented as mean ± SD (*n* = 12). (a‐e) Unpaired t‐test; **p < 0.01; *p < 0.05; ns: Not significant

We next aimed to test memory and spatial cognition in the Morris water maze (MWM) (Vorhees & Williams, 2006) in mice aged 4 months. To exclude changes in locomotion or behavior, we first assessed general activity as well as exploratory behavior in the open field test. GlcNAc feeding did not alter spontaneous locomotor activity or exploratory behavior as measured by distance, speed, and percentage of distance spent in the center of the open field at 6 months of age (Figure 3a,b; Figure S2a). Additionally, the analysis of the home cage activity in metabolic cages did not reveal differences caused by GlcNAc supplementation (Figure S2b). Thus, locomotion and exploratory behavior were not affected by GlcNAc treatment.

In the MWM, the animals were trained for five consecutive days, and after the last session, memory was assessed by removal of the hidden platform. During training, both distance and searching time (latency) decreased progressively (Figure S2c,d), while swimming speed remained constant (Figure S2e). These data suggest that the mice learned to find the hidden platform; however, there was no significant difference between GlcNAc‐fed animals compared with controls (Figure S2c‐e) during the training period. Still, GlcNAc‐supplemented male mice tended to swim shorter distances and to spend less time in the water from day 2 on (Figure S2c,d). During the test, there was no difference in the time that mice spent in the target quadrant upon removal of the platform (Figure 3c), strikingly, however, the latency of the first platform visit (defined as the time needed until the mice cross the area where the platform used to be located) was strongly decreased in GlcNAc‐fed males (Figure 3d). Furthermore, the number of platform visits (defined as the number of times the mice cross the area where the platform used to be located) was increased in GlcNAc‐fed male mice (Figure 3e). Overall, our observations suggest that GlcNAc feeding improves memory of young male mice.

### 2.4 HBP activation by overexpression of WT or G451E huGFAT1 does not influence body weight in mice

**FIGURE 4:**
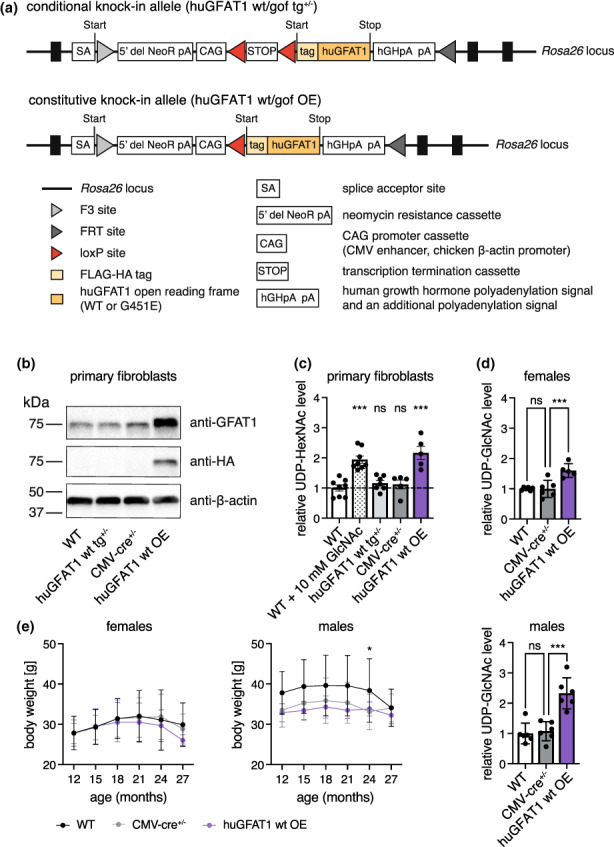
HBP activation by huGFAT1 wt OE does not influence body weight in mice. (a) Schematic representation of the transgene. The expression cassette was inserted in the *Rosa26* locus (conditional knock‐in allele). Upon cre‐mediated deletion of the transcription termination cassette, FLAG‐HA tagged human GFAT1 is expressed under the control of the chicken β‐actin promoter (constitutive knock‐in allele). (b) Western blot analysis of GFAT1 and HA expression in primary fibroblasts isolated from newborn mice (*n* = 1). β‐actin was used as loading control. (c) Relative UDP‐HexNAc levels in primary fibroblasts. (d) Relative UDP‐GlcNAc levels in hemibrain isolated from 3 months old control and huGFAT1 wt OE mice of both sexes. (c‐d) Data are presented as mean ± SEM (*n* ≥ 5). One‐way ANOVA, Dunnett's post‐test; ***p < 0.001; ns: Not significant. (e) Body weight of control and huGFAT1 wt OE mice of both sexes from 12 to 27 months of age. Data are presented as mean ± SD (females: *n* ≥ 4; Males: *n* ≥ 7; Details about the number of mice used at each time point is provided in Table S1). Body weight of female WT and CMV‐cre^+/−^ mice is also shown in figure S3d. Two‐way ANOVA, Dunnett's post‐test. Statistical significance was calculated compared with CMV‐cre^+/−^ mice at each time point; only significant changes are indicated. *p < 0.05

To corroborate the data obtained upon dietary GlcNAc supplementation, we pursued a parallel approach via genetic HBP activation by overexpressing N‐terminally FLAG‐HA tagged human GFAT1 (huGFAT1) in all mouse tissues. The *Rosa26* locus was engineered to contain an expression construct composed of a loxP‐flanked transcription termination cassette upstream of the huGFAT1 open reading frame (huGFAT1 wt tg^+/−^, Figure 4a). These mice were crossed with transgenic CMV‐cre^+/−^ females, to obtain huGFAT1 overexpressing animals (huGFAT1 wt OE, Figure 4a). The functionality of the cassette was confirmed by Western blot analysis (Figure 4b): In fibroblasts isolated from huGFAT1 wt tg^+/−^ newborn mice, HA expression was not detectable due to the lack of cre recombinase expression. Endogenous GFAT1 expression was comparable with WT and CMW‐cre^+/−^ fibroblasts. huGFAT1 wt OE fibroblasts, in which the GFAT1 transgene and cre recombinase were co‐expressed, displayed elevated GFAT1 and HA expression, demonstrating successful expression of the transgene. Accordingly, UDP‐HexNAc levels were comparable in WT, huGFAT1 wt tg^+/−^ and CMV‐cre^+/−^ fibroblasts and about 2‐fold elevated by GlcNAc supplementation and huGFAT1 wt overexpression (Figure 4c). As expected, huGFAT1 wt overexpression also resulted in elevated levels of UDP‐GlcNAc (Figure 4d) and UDP‐GalNAc (Figure S3a) in the brains of 3 months old male and female mice compared with brains of WT and CMV‐cre^+/−^ mice. Body weight analysis over 27 months indicated that there was no effect of huGFAT1 wt overexpression compared with CMV‐cre^+/−^ mice in both sexes (Figure 4e). However, cre expression slightly reduced body weight in male mice compared with WT controls (Figure 4e).

In addition, we generated huGFAT1 gain‐of‐function (gof) transgenic mice using the same strategy employed for the overexpression of wt huGFAT1. The GFAT‐1 G451E substitution is associated with *C. elegans* longevity (Denzel et al., 2014) and confers a drastically reduced sensitivity to UDP‐GlcNAc feedback inhibition (Ruegenberg et al., 2020). huGFAT1 G451E overexpression resulted in elevated levels of both UDP‐GlcNAc and UDP‐GalNAc in the brains of 3 months old male and female mice (Figure S3b,c). Body weight was not affected by huGFAT1 gof OE between 12 and 27 months of age compared with CMV‐cre^+/−^ mice in both sexes (Figure S3d). Together, these data suggest successful genetic HBP activation without detrimental effects on overall health as assessed by body weight analysis in huGFAT1 wt and gof OE mice. As we observed no relevant differences between huGFAT1 wt or G451E gof overexpression, we focused the remainder of the analyses on the huGFAT1 wt OE mice.

### 2.5 HBP activation by huGFAT1 WT OE does not influence coordination, neuromuscular function, memory, or lifespan

**FIGURE 5:**
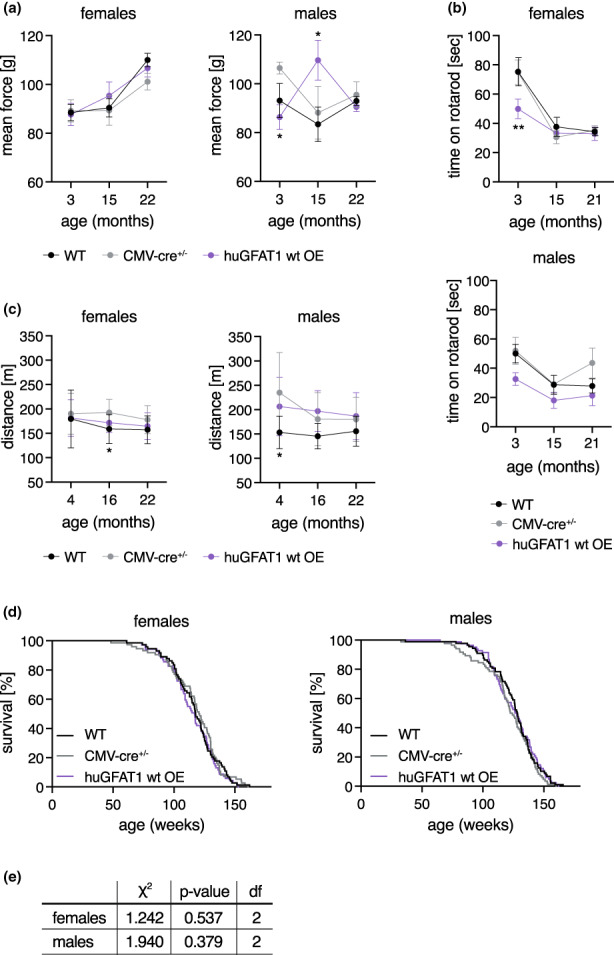
HBP activation by huGFAT1 wt OE does not affect fitness of mice. (a) Mean force measured in a grip strength test with two paws of control and huGFAT1 wt OE mice of both sexes at 3, 15, and 22 months of age. (b) Maximal time on a rotarod of control and huGFAT1 wt OE mice of both sexes at 3, 15, and 21 months of age. (a‐b) Data are presented as mean ± SEM (*n* ≥ 2). (c) Maximal distance on a treadmill of control and huGFAT1 wt OE mice of both sexes at 4, 16, and 22 months of age. Data are presented as mean ± SD (*n* ≥ 4). (a‐c) Two‐way ANOVA, Dunnett's post‐test. Statistical significance was calculated compared to CMV‐cre^+/−^ mice at each time point; only significant changes are indicated. **p < 0.01; *p < 0.05 (d) Lifespan analysis of control and huGFAT1 wt OE mice of both sexes (females: *n* ≥ 68; males: *n* ≥ 80). Survival of WT and CMV‐cre^+/−^ mice is also shown in figure S4b. (e) Cumulative incidence calculated by Gray's test based on the lifespan analysis shown in (d). Df: Degrees of freedom

To study coordination and basic neuromuscular function upon genetic HBP activation, experiments using rotarod and treadmill were performed, and grip strength was measured using all genotypes at three different time points (3–4, 15–16, and 21–22 months of age). In line with the results obtained using dietary GlcNAc supplementation, genetic HBP activation did not affect grip strength (Figure 5a, Figure S4a), rotarod performance (Figure 5b), or forced maximal endurance on a treadmill (Figure 5c). To assess the effect of genetic HBP activation on survival, lifespan experiments with transgenic mice of both sexes were performed. The survival of the huGFAT1 wt or gof OE mice was indistinguishable from the corresponding genetic controls (Figure 5d,e; Figure S4b,c).

**FIGURE 6:**
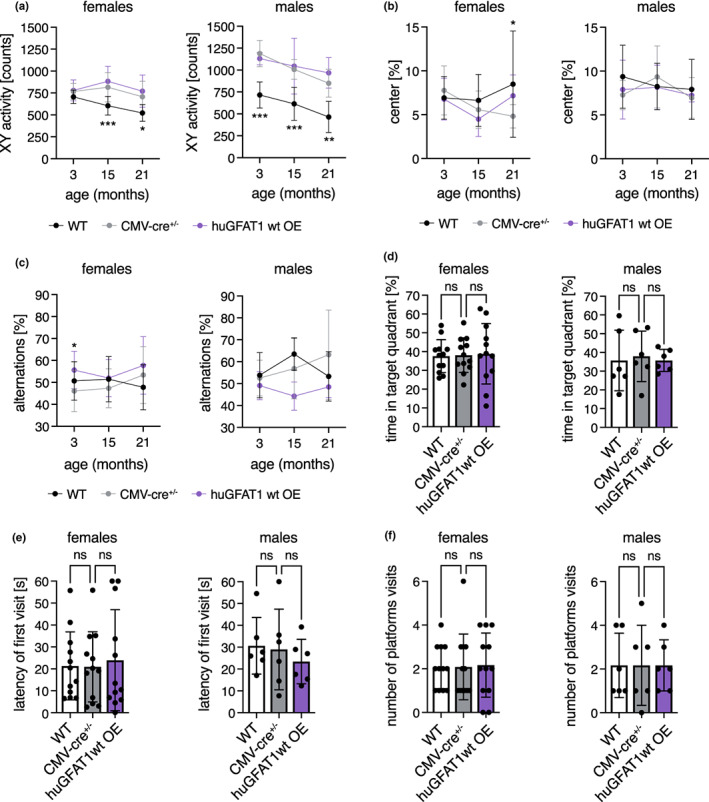
HBP activation by huGFAT1 wt OE does not influence spontaneous locomotor and exploratory behavior or memory of mice. (a) XY activity measured in the open field test of control and huGFAT1 wt OE mice of both sexes at 3, 15, and 21 months of age. (b) Percent of distance spent in the center of the open field of control and huGFAT1 wt OE mice of both sexes at 3, 15, and 21 months of age. (c) Percent of alternations measured in the Y maze test of control and huGFAT1 wt OE mice of both sexes at 3, 15, and 21 months of age. (a‐c) Data are presented as mean ± SD (*n* ≥ 4). Two‐way ANOVA, Dunnett's post‐test. Statistical significance was calculated compared to CMV‐cre^+/−^ mice at each time point; only significant changes are indicated. ***p < 0.001; **p < 0.01; *p < 0.05 (d) Percent of time spent in the target quadrant, (e) Latency of the first platform visit, and (f) Number of platform visits upon removal of the hidden platform in the Morris water maze test of control and huGFAT1 wt OE mice of both sexes at 4 months of age. (d‐f) Data are presented as mean ± SD (*n* ≥ 6). One‐way ANOVA, Dunnett's post‐test; ns: Not significant

We assessed both general locomotor activity and exploratory behavior in the open field test at 3, 15, and 21 months of age in both sexes. Activity and speed of the mice were influenced by cre expression, since CMV‐cre^+/−^ and huGFAT1 wt OE mice moved more and faster compared to WT controls (Figure 6a, Figure S5a). This effect was more pronounced in male mice than in females. Nevertheless, overall, genetic HBP activation had no effect on spontaneous locomotor activity. Exploratory behavior as measured by the percentage of distance spent in the center of the open field was similar in all genotypes (Figure 6b). Memory and learning were tested in both Y maze and MWM. In the Y maze, alternations, that is how often a mouse chooses to explore a new arm over the same arm, did not change among the different genotypes, suggesting no differences in exploratory behavior and spatial working memory (Figure 6c). The distance covered in the Y maze was comparable across all genotypes in females, while it was increased in males upon cre expression (Figure S5b). In the MWM, there was no difference in learning in 4 months old mice, since all genotypes of both sexes showed a similar improvement regarding distance and latency during the training sessions (Figure S5c,d). Speed was slightly decreased on day 5 compared with day 1, however, this effect was similar in all genotypes (Figure S5e). Memory, which was tested by removal of the platform, was unchanged, since the time spent in the target quadrant (Figure 6d), the latency of the first platform visit (Figure 6e), and the number of platform visits (Figure 6f) were similar in all genotypes. Altogether, and in contrast to dietary GlcNAc supplementation, genetic HBP activation did not improve memory in mice. Overall, results obtained using the genetic model support the conclusion that HBP activation does not have adverse effects in mice. While lifespan was not affected in any of the HBP activation regimens tested, GlcNAc supplementation improved memory in young male mice.

## 3 DISCUSSION

In this study, we investigated the link between chronic HBP activation, healthspan, and lifespan in mice. We delineated the effect of HBP activation on general behavior, memory, and survival. Importantly, we showed that dietary GlcNAc supplementation or GFAT1 overexpression did not have negative side effects on the overall health of mice. While fitness, locomotion, behavior, and survival were not changed by HBP activation, we observed memory improvement in young male mice fed with GlcNAc, suggesting a beneficial effect of HBP activation in mice.

Dietary GlcNAc is rapidly absorbed and enters the systemic circulation when fed via the drinking water: experiments using ^13^C_6_‐GlcNAc indicate that GlcNAc peaks in the serum after 30 min of gavage and is cleared from the circulation after 2–3 h; by this time, UDP‐^13^C_6_‐GlcNAc can be detected in liver, kidney, and spleen (Ryczko et al., 2016). ^13^C_6_‐GlcNAc, orally supplemented at a concentration of 0.1%, has been shown to cross the blood brain barrier after feeding 8 week old mice for 3 days: LC–MS/MS analysis identified UDP‐[U^13^C]‐HexNAc in the brain of adult females (Sy et al., 2020). These data indicate that GlcNAc supplied via the drinking water contributes to the UDP‐GlcNAc pool in brain and liver. While we showed in previous studies that GlcNAc supplementation increases UDP‐GlcNAc levels in worms and in cells (Denzel et al., 2014; Horn et al., 2020), the steady‐state levels of UDP‐HexNAc were not increased in brain or liver of mice fed with 1% GlcNAc after 7 months of supplementation. Previously, it has been shown that GlcNAc feeding elevates hepatic UDP‐HexNAc levels by around 25% (Ryczko et al., 2016). Given this relatively small effect and the fast clearance of GlcNAc from the circulation, the timing of sample collection might have influenced our results: The mice were sacrificed during the day when they drink less. Moreover, we analyzed steady‐state UDP‐HexNAc levels; thus, we cannot exclude elevated HBP flux upon dietary GlcNAc supplementation. Ubiquitous overexpression of wt GFAT1 resulted in elevated steady‐state levels of UDP‐HexNAc in the brain of mice aged 3 months. Overexpression of the G451E gain‐of‐function GFAT1 variant, which displays reduced sensitivity to UDP‐GlcNAc feedback inhibition (Ruegenberg 2020), activated the HBP to a similar extent as wt GFAT1 OE. Thus, it is likely that the N‐terminal tag interfered with GFAT1 activity and/or the allosteric feedback regulation of the enzyme, the former being previously described (Olchowy et al. 2006). Nevertheless, the mice had around 2‐fold increased UDP‐HexNAc levels, demonstrating successful HBP activation in our genetic model.

Dietary GlcNAc supplementation has been previously shown to increase body weight: post‐weaning C57BL/6 male mice fed with 0.5%–1.5% GlcNAc in the drinking water had a surge in body weight after around 3 months of feeding, with a concomitant increment in hepatic UDP‐GlcNAc levels (Ryczko et al., 2016). In contrast, 1% GlcNAc in the drinking water did not significantly change body weight in males up to 30 months of age in our study. However, females fed with 1% GlcNAc showed significantly increased body weight up to 10 months of age; later in life, body weight was elevated without reaching statistical significance. Interestingly, 9 months old females consumed more GlcNAc‐containing water; however, they did not display significantly elevated UDP‐HexNAc levels. Thus, GlcNAc intake rather than UDP‐HexNAc levels correlated with body weight. This notion is supported by the findings obtained using the transgenic animals: while wt or GFAT1 G415E overexpression resulted in elevated brain UDP‐HexNAc levels, body weight did not differ from control CMV‐cre^+/−^ mice. Previously, GFAT1 overexpression in the liver has been associated with increased body weight (Veerababu et al., 2000). However, since the increase in hepatic UDP‐GlcNAc levels in those transgenic mice is comparable with the extent of HBP activation seen in our genetic model, it is unlikely that elevated UDP‐GlcNAc levels are the underlying cause of the increased weight.

HBP activation has been linked to impaired insulin signaling: adipocytes treated with GlcN become insulin resistant (Marshall et al., 1991) and rats infused with GlcN also exhibit insulin resistance (Virkamaki, 1997), similarly to GlcN‐treated mice (Weimer et al., 2014). Studies in humans have shown that 1.5 g of GlcN fed orally for 6 weeks augmented the risk for diabetes in adults with previous poor insulin sensitivity (Pham et al., 2007). In our experiments, GlcNAc feeding did not alter the response to insulin up to 20 months of age. In line with our results, Ryczko et al. (2016) found that mice fed with 0.05% GlcNAc for 23 weeks showed no difference to the controls in the glucose tolerance test. Additionally, rats fed 5% GlcNAc for 1 year (pellet‐based supplementation) did not exhibit changes in the basal levels of serum glucose (Takahashi et al., 2009). Overall, current data suggest that dietary GlcN‐mediated HBP activation augments the risk for insulin resistance from mice to humans, whereas dietary GlcNAc supplementation does not cause adverse effects. Therefore, GlcNAc feeding, in contrast to GlcN supplementation, might be safe in people and could be used to treat certain diseases, for example osteoarthritis, a condition that is currently medicated with oral GlcN (Block et al., 2010).

In nematodes, HBP activation by GlcNAc supplementation and GFAT‐1 gof mutations extends survival (Denzel et al., 2014). In contrast, survival of mice fed with 1% GlcNAc was similar to controls in this study. One possible explanation is that the level of HBP activation achieved in our experiments might not be sufficient to extend lifespan. However, we also did not observe an effect on survival using our genetic model in which UDP‐GlcNAc levels were 2‐fold elevated. Thus, while genetic HBP activation results in longevity in *C. elegans*, it was not sufficient to extend lifespan in mice. These data suggest that the lifespan‐modulating mechanisms of HBP activation in the worm are not conserved. Of note, *Drosophila melanogaster* flies with increased HBP flux achieved by GlcN feeding display increased mortality (Na et al., 2013). In worms and mice, GlcN feeding extends lifespan in a manner that is uncoupled from the HBP: GlcN supplementation does not increase the levels of UDP‐GlcNAc in mouse liver and silencing of the HBP enzyme PGM3 by RNA interference does not impair GlcN‐mediated lifespan extension in worms (Weimer et al., 2014). Overall, these data demonstrate that a modest UDP‐GlcNAc elevation does not extend survival in mice while treatments with upstream aminosugars such as GlcN achieve beneficial effects through HBP‐independent mechanisms.

Putative loss‐of‐function mutations in *GFAT1* are associated with congenital myasthenic syndrome, which is characterized by defective neuromuscular junctions (Senderek et al., 2011). Consistently, activating the HBP pathway might have beneficial effects on neuromuscular function. Indeed, GlcN supplementation has been described to improve motor coordination in mice (de La Rosa et al., 2022). However, we did not observe differences in coordination and fitness upon GlcNAc feeding or genetic HBP activation. While spontaneous locomotor activity and exploratory behavior were unchanged in GlcNAc‐fed mice compared with controls, GlcNAc‐fed young males exhibited memory improvement. Of note, it has been previously shown that scopolamine‐induced spatial learning and memory deficits could be alleviated by GlcN injections in male Wistar rats in the MWM (Jamialahmadi et al., 2013). Furthermore, GlcN had neuroprotective effects in a rat middle cerebral artery occlusion model (Hwang et al., 2010). Together, these data and our results suggest that HBP supplementation might have beneficial effects on brain function and memory. To date, it is unknown which mechanisms underly the neuroprotective effects. In contrast to GlcNAc feeding, GFAT1 overexpression did not enhance learning and memory formation compared with controls. These data suggest that the GlcNAc‐mediated effect on memory might be independent of HBP activity. Based on the positive effect on spatial learning and memory, it has been suggested that GlcN may be used for prevention or treatment of neurodegenerative diseases (Dalirfardouei et al., 2016). Our data demonstrate that GlcNAc treatment has similar beneficial effects, while causing no adverse reactions in mice. Thus, we provide evidence that GlcNAc, rather than GlcN, should be tested as potential treatment for neurodegenerative diseases in the future.

In sum, this study dissects the effects of dietary GlcNAc supplementation and genetic HBP activation on murine health, behavior, memory, and lifespan. Despite being linked to diabetes, obesity, and cancer, these interventions did not have adverse effects in mice. Instead, we show that GlcNAc feeding increases memory formation of young male mice. Of note, this effect might be independent of HBP activity since we did not observe changes in memory upon genetic HBP activation. In the future, HBP‐independent effects of GlcNAc supplementation should be tested in the context of learning and memory. Based on these data, GlcNAc treatment should be considered as a viable therapeutic alternative to GlcN supplementation as it might lead to fewer adverse effects.

### 3.1 Limitations of the study

This study was performed using 1% GlcNAc in the drinking water, which did not elevate the steady‐state levels of UDP‐HexNAc in the analyzed tissues. Thus, the HBP might not be sufficiently activated using this paradigm. Whether higher GlcNAc concentrations would affect general health and lifespan remains an open question.

Additionally, the beneficial effect of GlcNAc feeding on learning and memory could not be detected in the genetic model of HBP activation. Hence, it is likely that GlcNAc treatment is promoting memory formation in an HBP‐independent manner, but the molecular mechanism of GlcNAc action remains unclear.

## 4 EXPERIMENTAL PROCEDURES

### 4.1 Mouse husbandry

C57BL/6J mice were housed on a 12:12 h light: dark cycle with ad libitum access to food under pathogen‐free conditions in individually ventilated cages. Animal care and experimental procedures were in accordance with the institutional and governmental guidelines.

### 4.2 GlcNAc feeding

The mice were fed with control water or water containing 1% GlcNAc (w/v) from 8 weeks of age. GlcNAc was provided by Wellesley Therapeutics Inc., Toronto, Canada.

### 4.3 Generation of transgenic mice

Generation of transgenic GFAT1 mice was performed by Taconic Biosciences (Cologne, Germany). An expression cassette was inserted in the *Rosa26* locus using recombination‐mediated cassette exchange in embryonic stem cells. The cassette encodes a loxP‐flanked transcription termination cassette upstream of the human GFAT1 (huGFAT1) open reading frame (conditional knock‐in allele, huGFAT1 wt/gof tg^+/−^). Upon cre‐mediated deletion of the transcription termination cassette, huGAFT1 is expressed under the control of the chicken b‐actin promoter, resulting in its overexpression (constitutive knock‐in allele, huGFAT1 wt/gof OE). huGFAT1 is N‐terminally tagged with FLAG‐HA (HA: hemagglutinin; for further information, see Figure 4).

### 4.4 Tissue collection

Mice were sacrificed by cervical dislocation. Brains from 3 to 9 months old mice were dissected. Cerebral hemispheres were snap frozen in liquid nitrogen and stored at −80°C.

### 4.5 Lifespan analysis

Lifespan analysis was performed with 69 female and 82 male mice per genotype/condition. Mice were monitored regularly and euthanized when necessary, according to a pre‐defined score sheet. The lifespan analysis comprises all mice that died a natural death or were euthanized. The body weight of the mice was analyzed every other week (GlcNAc feeding) or every 3 months (huGFAT1 wt/gof OE). To exclude an effect on lifespan, no other experiments were performed with these mice. The 19 months' time point was not recorded for female mice treated with GlcNAc and was therefore not included in Figure 1g.

### 4.6 Metabolic cages

Indirect metabolic analyses were performed in singly housed 3 and 9 months old mice for 48 h using metabolic cages (Phenomaster, TSE Systems). Mice were habituated to the cages for 24 h before measurements. Analysis of the air before and after passing the cages allowed calculation of oxygen consumption and carbon dioxide production. These values were used to determine the metabolic respiratory quotient. Spontaneous locomotor activity, as well as food and water consumption of the mice were monitored.

### 4.7 Glucose and insulin tolerance tests

Glucose and insulin tolerance was determined in 4, 10, and 20 months old mice upon GlcNAc feeding. For the glucose tolerance test, mice were fasted for 16 h with full access to drinking water. 2 g glucose per kg body weight were injected intraperitoneally. To monitor blood glucose concentration, a blood sample was taken before and 15, 30, 60, and 120 min after glucose injection. For the insulin tolerance test, 0.75 U insulin per kg body weight were injected intraperitoneally. To monitor blood glucose concentration, a blood sample was taken before and 15, 30, and 60 min after the insulin injection. Blood glucose concentration was measured using an automatic glucose monitor (Accu‐Check Aviva, Roche).

### 4.8 Grip strength measurements

For grip strength measurements, a mouse held a trapeze while being pulled backward until the pulling force exceeded grip strength (2 paws). Maximal grip strength was recorded automatically. Alternatively, the mouse was placed on a grid and the grasping applied by the mouse while being pulled backwards was measured (4 paws).

The tests were repeated five times per mouse per day and the mean was plotted.

### 4.9 Open field

Mice were placed in a 50 × 50 × 40 cm big box for 10 min. Spontaneous locomotor and explorative activity, as well as the speed were analyzed by tracking movement of the mice.

### 4.10 Rotarod

Mice were placed on the rotarod and rotation speed of the rod constantly increased from 5 to 40 revolutions per min within 5 min. The time until the mice fell of the rod was measured. The experiment was performed twice per day on two or four consecutive days for GlcNAc feeding and huGFAT1 wt OE mice, respectively. The average time on the rod of the two runs on the last day of the experiment was plotted.

### 4.11 Treadmill

After an adaption phase of 5 min, the treadmill was started with a speed of 0.1 m/s. This speed was maintained for 10 min before it constantly increased to 1.3 m/s within 60 min. An electric shock of 0.3 mA was given when the mouse stayed at the end of the treadmill for more than 2 s. The experiment was stopped when a mouse received three consecutive shocks. The cumulative distance was analyzed for each mouse.

### 4.12 Y maze

The mouse was placed in one arm of the Y maze for 5 min. The position of the mouse was tracked. Distance and alternations were measured for each mouse. The alternations describe how often a mouse chooses to explore a new region over the same region.

### 4.13 Water maze

Water maze analyses were performed with 4 months of age. The mice were placed in a basin filled with stained water, in which a platform was hidden 1–2 cm below the surface of the water. The mice will learn to find the hidden platform during training sessions on five consecutive days. The mice performed four trials per day, with a maximum duration of 60 s per trial. The average of the four trials is plotted for each day. After the training on Day 5, the platform was removed. During this final test, the mice were placed in the basin for 60 s and their preference for the quadrant which previously contained the platform, as well as the latency of the first visit and the number of visits of the former platform area was analyzed.

### 4.14 Isolation of mouse genomic DNA from ear clips

Ear clips were taken by the Comparative Biology Facility at the Max Planck Institute for Biology of Ageing (Cologne, Germany) at weaning age (3–4 weeks of age). The ear clips were lysed in 150 μl ddH2O and 150 μl directPCR Tail Lysis reagent (Peqlab) containing 3 μl proteinase K (20 mg/ml in 25 mM Tris–HCl, 5 mM Ca2Cl, pH 8.0, Sigma‐Aldrich) over night (maximum 16 h) at 56°C. Proteinase K was inactivated at 85°C for 45 min. The lysis reaction was used for PCR without further processing. For genotyping of newborn pups, the tail tip was lysed in 300 μl directPCR Tail Lysis 463 reagent.

### 4.15 Genotyping PCR

For genotyping of mouse, genomic DNA DreamTaq DNA polymerase (ThermoFisher Scientific) was used. Primer information is summarized below (Table 1):

**TABLE 1:** Primer mixes used for mouse genotyping PCRs

PCR	Primer name	T_A_ [°C]	Size [bp]	GenBank no.	BLAST E‐value	BLAST Next/*first E‐value
Cre	Cre_fwd Cre_rev Il2_fwd Il2_rev	60	700 300	binds transgene binds transgene AH001969.2 AH001969.2	n.d. n.d. 2*10^−4^ 6*10^−5^	1.20* 1.20* 0.71 0.83
hu GFAT1	huGFAT1_fwd huGFAT1_rev Il2_fwd Il2_rev	64	566 300	binds to FLAG‐HA NM_001244710.2 AH001969.2 AH001969.2	n.d. 1*10^−4^ 2*10^−4^ 6*10^−5^	3.60* 0.40 0.71 0.83
hu GFAT1 _R26	3224_35 1114_1 1114_2	60	744 (tg) 299 (wt)	binds cassette NC_000072.7 NC_000072.7	n.d. 1*10^−5^ 1*10^−4^	1.20* 2.10 1.80

*Note*: The annealing temperature (T_A_), size of 469 the amplicon, GenBank accession number, and BLAST E‐values are indicated for each primer. N.d.: not determined.

The presence of the cre‐transgene was determined using Cre_fwd (GCCAGCTAAACATGCTTCATC) and Cre_rev (ATTGCCCCTGTTTCACTATCC). The primer targeting huGFAT1 (huGFAT1_fwd CGGTGGAGGTTACCCATACG; huGFAT1_rev CGAGCTTGGCAATTGTCTCTG) detected the presence of the transgene without distinction of zygosity. The product amplified using Il2_fwd (CTAGGCCACAGAATTGAAAGATCT) and Il2_rev (GTAGGTGGAAATTCTAGCATCATCC) served as internal control (interleukin 2) for huGFAT1 and cre‐transgene detection. Using the huGFAT1_R26 PCR (3224_35 TTGGGTCCACTCAGTAGATGC; 1114_1 CTCTTCCCTCGTGATCTGCAACTCC; 1114_2 CATGTCTTTAATCTACCTCGATGG), the wildtype and the targeted *Rosa26* locus could be distinguished.

### 4.16 Isolation and maintenance of primary fibroblasts

For fibroblast isolation, newborn mice (P0‐P3, both sexes) were sacrificed by decapitation. The corpus was incubated in 50% betaisodona/PBS (Mundipharma GmbH) for 30 min at 4°C before being washed in different solutions for 2 min each: PBS (ThermoFisher Scientific), 0.1% octenidin in ddH_2_O (Serva Electrophoresis), PBS, 70% ethanol, PBS, antibiotic‐antifungal‐solution in PBS (ThermoFisher Scientific). Tail and legs were removed. Complete skin was separated from the body and incubated in 2 ml dispase II solution (5 mg/ml in 50 mM HEPES/KOH pH 7.4, 150 mM NaCl; Sigma‐Aldrich) over night at 4°C. The epidermis was separated from the dermis as a sheet. The dermis was minced into small pieces using scalpels and transferred to a falcon tube containing collagenase (400 U/ml in 50 mM Tris base, 5 mM CaCl_2_, pH 7.4; Sigma‐Aldrich). The samples were incubated at 37°C for 1.5 h and mixed regularly. Next, the suspension was filtered through a 70 μm cell strainer, which was washed with DMEM afterwards. The cells were centrifuged for 10 min at 1000 rpm. The pellet was resuspended in DMEM (4.5 g/L glucose, 10% fetal bovine serum, and penicillin/streptavidin; all ThermoFisher Scientific) and the cells were seeded on non‐coated tissue culture plates. The cells were grown at 37°C in 5% CO_2_.

### 4.17 Western blot analysis

Protein concentration of cell lysates was determined using the Pierce™ BCA protein assay kit according to manufacturer's instructions (ThermoFisher Scientific). Samples were subjected to SDS‐PAGE and blotted on a nitrocellulose membrane. The following antibodies were used in 5% low‐fat milk (Carl Roth) or 1% bovine serum albumin (BSA; Carl Roth) in TBS‐Tween buffer (25 mM Tris base, 150 mM NaCl, 2 mM KCl, pH 7.4; 0.05% Tween‐20 (w/v)) over night at 4°C: GFPT1 (rabbit, Abcam, EPR4854, 1:1.000 in BSA), hemagglutinin (HA; rat, Roche Diagnostics GmbH, 3F10, 1:1.000 in milk), b‐actin (mouse, Cell Signaling Technologies, 8H10D10, 1:25.000 in milk). After incubation with HRP‐conjugated secondary antibody (Invitrogen, 1:5000), the blot was developed using ECL solution (Merck Millipore). Bands were detected on a ChemiDoc MP Imaging System (Bio‐Rad Laboratories).

### 4.18 Metabolite analysis

#### 4.18.1 Determination of UDP‐HexNAc levels

Brains were collected from 9 months old mice, cut in half, and snap frozen in liquid nitrogen. For metabolite extraction, 250 μl ddH2O were added to the hemibrains and the tissue was disrupted using a dounce homogenizer. The samples were subjected to four freeze/thaw cycles (liquid nitrogen/37°C water bath). Next, the protein concentration was measured using the Pierce™ BCA protein assay kit (ThermoFisher Scientific). 200 μl with a protein concentration of 1 μg/μl were mixed with 1 ml chloroform: methanol (1:2) and incubated on a nutator mixer for 1 h at RT. After centrifugation for 5 min at full speed, the supernatant was transferred to a glass vial. The liquid was evaporated in an EZ‐2 Plus Genevac centrifuge evaporator (SP Scientific) with the following settings: time to final stage 15 min, final stage time 4 h, low boiling point mixture. After evaporation, the samples were stored at −20°C until further use.

Absolute UDP‐HexNAc levels were determined using an Acquity UPLC connected to a Xevo TQ Mass Spectrometer (both Waters) and normalized to total protein content. The measurements and subsequent analysis were performed as previously described (Denzel et al., 2014).

#### 4.18.2 Determination of UDP‐GlcNAc and UDP‐GalNAc levels

Brains were collected from 3 months old mice, cut in half, and snap frozen in liquid nitrogen. Tissue was disrupted using a TissueLyser II (Qiagen) at 20–25 Hz. The powder was transferred to a fresh tube and subjected to metabolite extraction.

Metabolite extraction was performed using 80% methanol. After vortexing, the samples were incubated at −20°C for 30 min. Afterward, samples were incubated on an orbital mixer at 5°C for 30 min. The samples were centrifuged for 5 min at full speed and 4°C. The supernatant was transferred to a fresh tube, and the pellet was used for protein extraction with 0.5% SDS. The supernatant was evaporated in a SpeedVac concentrator at 25°C.

The metabolite analysis was conducted using a Dionex ICS‐5000 anion exchange chromatography (ThermoFisher Scientific). Separation was performed with a Dionex Ionpac AS11‐HC column (2 × 250 mm, 4 μm particle size, Thermo Fisher) at 30°C. A guard column, Dionex Ionpac AG11‐HC b (2 × 50 mm, 4 μm particle size, ThermoFisher Scientific), was placed before the separation column. The eluent (KOH) was generated by a KOH cartridge using ddH_2_O. A gradient was used for the separation at a flow rate of 0.380 ml/min: 0–8 min 30 mM KOH, 8–12 min 35–100 mM KOH, 12–15 min 100 mM KOH, 15–19 min 30 mM KOH. A Dionex suppressor AERS 500 (2 mm) was used for the exchange of KOH and operated with 95 mA at 17°C. The suppressor pump flow was set to 0.6 ml/min. Samples were diluted in ddH_2_O and injected from a tempered autosampler (8°C) using full loop mode (10 μl). The Dionex ICS‐5000 was connected to a XevoTM TQ mass spectrometer (Waters) and operated in negative ESI MRM (multi reaction monitoring) mode. The source temperature was set to 150°C, the desolvation temperature was set to 350°C, and desolvation gas was set to 650 L/h, while cone gas was set to 50 L/h. The MRM transition 606.10, 158.80 was used for quantification of UDP‐GlcNAc and UDP‐GalNAc. An external standard calibration curve was prepared from 50 to 1000 ng/ml UDP‐GlcNAc and UDP‐GalNAc. Data were analyzed using the MassLynx and TargetLynx software (Waters).

### 4.19 Statistical analysis

Data are presented as mean ± SEM or mean ± SD. Dots represent biological replicates; each biological replicate is an independent mouse. Statistical significance was calculated using GraphPad Prism 8 (GraphPad Software) except for the cumulative incidence, which was calculated using the R package cmprsk. The statistical test used is indicated in the respective figure legend. Significance levels are * *p* < 0.05, ** *p* < 0.01, *** *p* < 0.001 versus the respective control.

AUTHOR CONTRIBUTIONS

Kira Allmeroth, Matías D. Hartman, Andrea Mesaros, and Martin S. Denzel designed the research. Kira Allmeroth and Matías D. Hartman wrote the manuscript. Martin Purrio and Andrea Mesaros performed mouse phenotyping. Kira Allmeroth performed all other experiments.

## CONFLICT OF INTEREST

The authors declare no potential conflict of interests.

## Supporting information


**Appendix S1** Supplementary Information

## Data Availability

The raw data that support the findings in the figures of this study are available from the corresponding author upon reasonable request.
