# Supplementary material for: Hexosamine pathway activation improves memory but does not extend lifespan in mice
Source: Aging Cell. 2022 Sep 19;21(10):e13711. doi: 10.1111/acel.13711 (PMC9577955; doi:10.1111/acel.13711)
Supplement: Supplementary file 1 — Appendix S1 Supplementary Information [file ACEL-21-e13711-s001.docx]

# Supplementary figures

**A** females males **^B^** females males


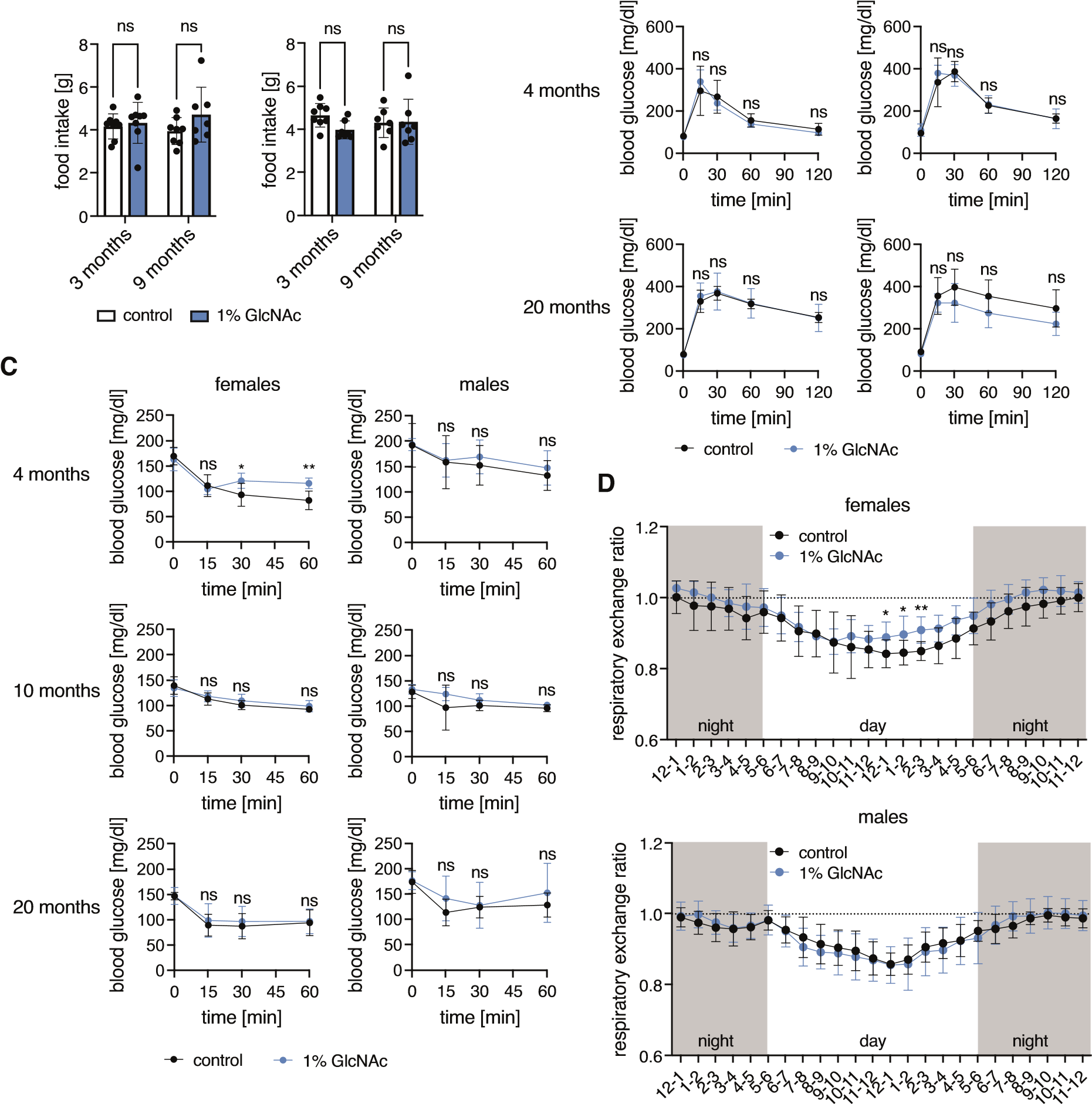


**Figure S1: GlcNAc supplementation does not influence food intake or insulin tolerance in mice.** (A) Food intake of control (white) and GlcNAc-treated mice (blue) of both sexes at 3 and 9 months of age measured in the metabolic cages. Data are presented as mean ± SD (n³7). Two-way ANOVA, Tukey’s post-test; ns: not significant (B) Blood glucose concentration at 0 (fasting), 15, 30, 60, and 120 min after intraperitoneal injection of glucose solution (2 g/kg body weight) of control (black) and GlcNAc-treated mice (blue) of both sexes at 4 and 20 months of age. (C) Blood glucose concentration before (0), and 15, 30, and 60 min after intraperitoneal injection of insulin (75 U/kg body weight) of control (black) and GlcNAc-treated mice (blue) of both sexes at 4, 10, and 20 months of age. (B-C) Data are presented as mean ± SD

825 (n³6). Multiple unpaired t-tests; ** p<0.01; * p<0.05; ns: not significant (D) Respiratory 826 exchange ratio (CO_2_ production/O_2_ consumption) of control (black) and GlcNAc827 treated mice (blue) of both sexes during the day and at night (gray) at 9 months of age 828 measured in the metabolic cages. Data are presented as mean ± SD (n³7). Multiple 829 unpaired t-tests; ** p<0.01; * p<0.05; only significant changes are indicated.

**A** females males **B** females males ns ns ns ns ns ns

control

1

% GlcNAc

0

10

20

30

40

speed [cm/s]

control

1

% GlcNAc

0

10

20

30

40

speed [cm/s]

day

night

0

5000

10000

15000

XY activity [counts]

day

night

0

5000

10000

15000

XY activity [counts]

control

% GlcNAc

1


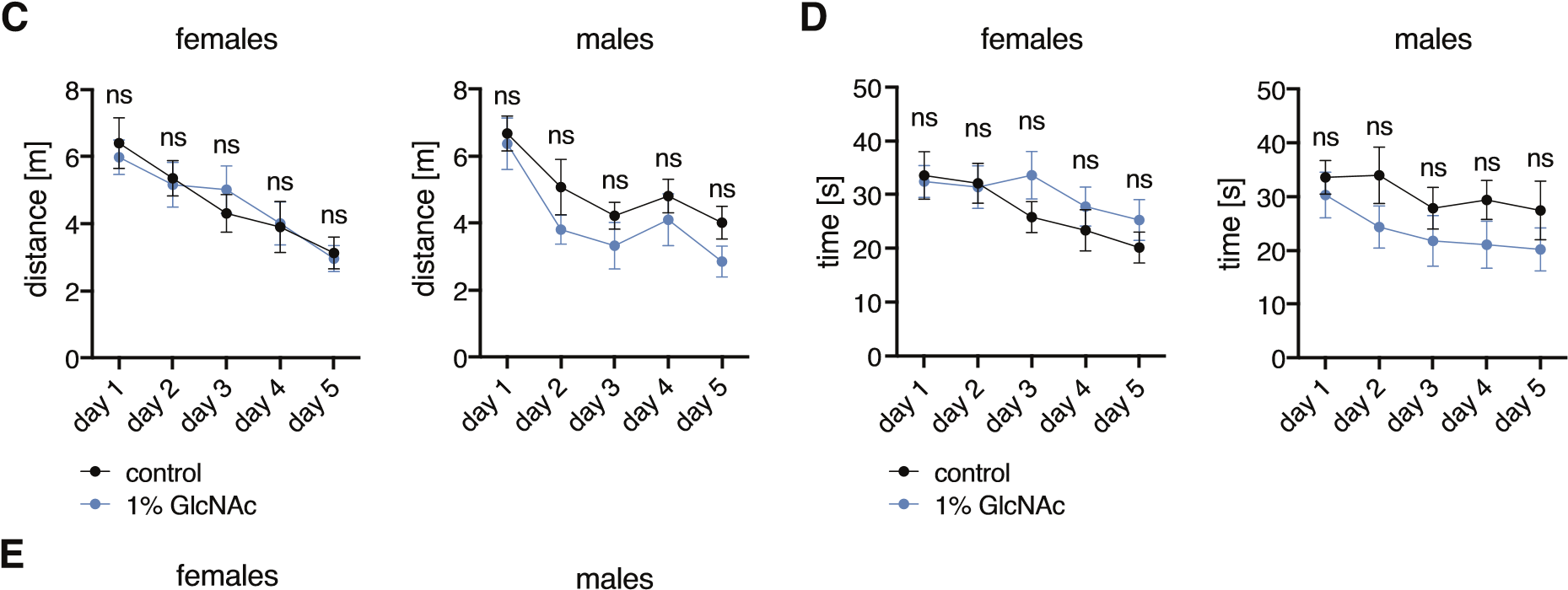


day 1

day 2

day 3

day 4

day 5

0

5

10

15

20

25

speed [cm/s]

ns

ns

ns

ns

ns

day 1

day 2

day 3

day 4

day 5

0

5

10

15

20

25

speed [cm/s]

ns

ns

ns

ns

ns

control

1

% GlcNAc

**Figure S2: GlcNAc supplementation does not influence spontaneous locomotor behavior or learning in mice.** (A) Speed of control (white) and GlcNAc-treated mice (blue) of both sexes at 6 months of age measured in the open field test. Data are presented as mean ± SD (n³12). Unpaired t-test; ns: not significant (B) XY activity of control (white) and GlcNAc-treated mice (blue) of both sexes during the day and at night (gray) at 3 months of age measured in the metabolic cages. Data are presented as mean ± SD (n³7). Two-way ANOVA, Tukey’s post-test; ns: not significant. (C) Distance and (D) Time until the mice reached the hidden platform, and (E) Swimming speed of control (black) and GlcNAc-treated mice (blue) of both sexes at 4 months of age during the Morris water maze training. (C-E) Data are presented as mean ± SEM. (n=12). The mean of four trials per day is plotted. Multiple unpaired t-tests; ns: not significant.

**A** females _males_ **^B^** females males

WT

CMV-cre

+

-

/

huGFAT1 wt OE

relative UDP-GalNAc level

WT

CMV-cre

+

/

-

huGFAT1 wt OE

relative UDP-GalNAc level

WT

CMV-cre

/

+

-

huG

FAT1 gof OE

relative UDP-GlcNAc level

WT

CMV-cre

/

+

-

huGFAT1 gof OE

relative UDP-GlcNAc level

0

1

2

3

0

1

2

3

ns

***

ns

***

0

0.5

1.0

1.5

2.0

2.5

3.0

3.5

ns

***

0

0.5

1.0

1.5

2.0

2.5

3.0

3.5

ns

*

**C** females males **D**


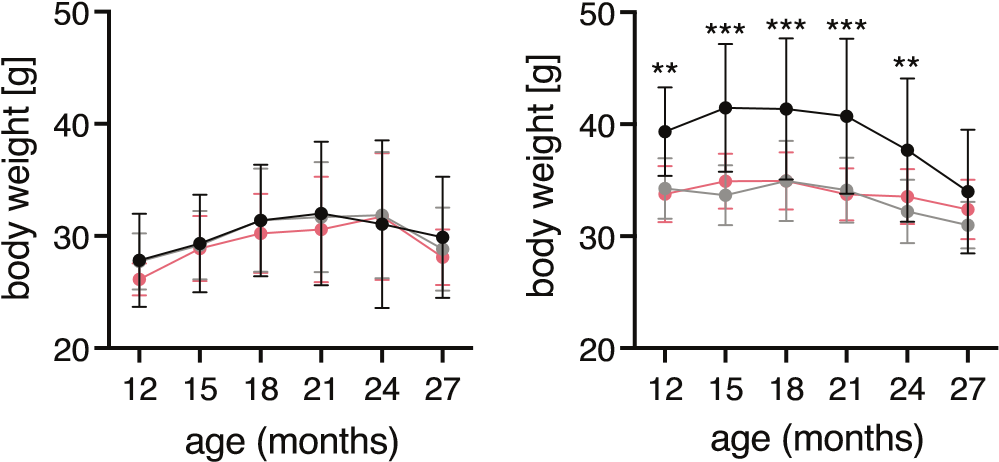
 _ns ns_ females males

WT

CMV-cre

+

-

/

huGFAT1 gof OE

relative UDP-GalNAc level

WT

CMV-cre

-

+

/

huGFAT1 gof OE

relative UDP-GalNAc level

0

1

2

3

0

1

2

3

ns

**

WT CMV-cre^+/-^  huGFAT1 gof OE

**Figure S3: HBP activation by huGFAT1 wt/gof OE in mice.** (A) Relative UDP-GalNAc levels in hemibrain isolated from 3 months old control and huGFAT1 wt OE mice of both sexes. (B) Relative UDP-GlcNAc levels in hemibrain isolated from 3 months old control and huGFAT1 gof OE mice of both sexes. (C) Relative

UDP-GalNAc levels in hemibrain isolated from 3 months old control and huGFAT1 gof OE mice of both sexes. (A-C) Data are presented as mean ± SEM. One-way ANOVA, Dunnett’s post-test; *** p<0.001; ** p<0.01; * p<0.05; ns: not significant. (D) Body weight of control and huGFAT1 gof OE mice of both sexes from 12 to 27 months of age. Data are presented as mean ± SD (females: n³6; males: n³7; details about the number of mice used at each time point is provided in Table S2). Body weight of female WT and CMV-cre^+/-^ mice is also shown in Figure 4e. Two-way ANOVA, Dunnett’s posttest. Statistical significance was calculated compared to CMV-cre^+/-^ mice at each time

point; only significant changes are indicated. *** p<0.001; ** p<0.01 **A**

age (months)

mean force [g]

females

age (months)

mean force [g]

males

WT

CMV-cre

+

/

-

huGFAT1 wt OE

15

22

3

80

100

120

140

160

180

*

22

3

15

80

100

120

140

160

180

**

**B**

males

females

age (weeks)

age (weeks)

survival [%]

survival [%]

WT

OE

FAT1 gof

huG

V-cre

CM

^-^

^/^

^+^

WT

OE

huG

FAT1 gof

V-cre

CM

^-^

^+^

^/^

0

150

50

100

0

20

40

60

80

100

0

50

100

150

0

20

40

60

80

100

**C**

|  | χ2 | p-value | df |
| --- | --- | --- | --- |
| females | 0.069 | 0.966 | 2 |
| males | 1.401 | 0.496 | 2 |

**Figure S4: Genetic HBP activation does not affect fitness of mice.** (A) Mean force measured in a grip strength test with four paws of control and huGFAT1 wt OE mice of both sexes at 3, 15 and 22 months of age. Data are presented as mean ± SD (n³4). Two-way ANOVA, Dunnett’s post-test. Statistical significance was calculated compared to CMV-cre^+/-^ mice at each time point; only significant changes are indicated. ** p<0.01; * p<0.05 (B) Lifespan analysis of control and huGFAT1 gof OE mice of both sexes (females: n³68; males: n³80). Survival of WT and CMV-cre^+/-^ mice is also shown in Figure 5d. (C) Cumulative incidence calculated by Gray’s test based on the lifespan analysis shown in (B). df: degrees of freedom.

| 3 15 21 age (months) | 3 15 21 age (months) | 3 15 21 age (months) | 3 15 21 age (months) |
| --- | --- | --- | --- |
| WT CMV-cre^+/-^ | huGFAT1 wt OE | WT CMV-cre^+/-^ | huGFAT1 wt OE |

**A** females males **B** females males

0

10

20

30

40

speed [cm/s]

0

10

20

30

40

speed [cm/s]

***

**

***

***

**

0

10

20

30

40

distance [m]

0

10

20

30

40

distance [m]

*

***

***


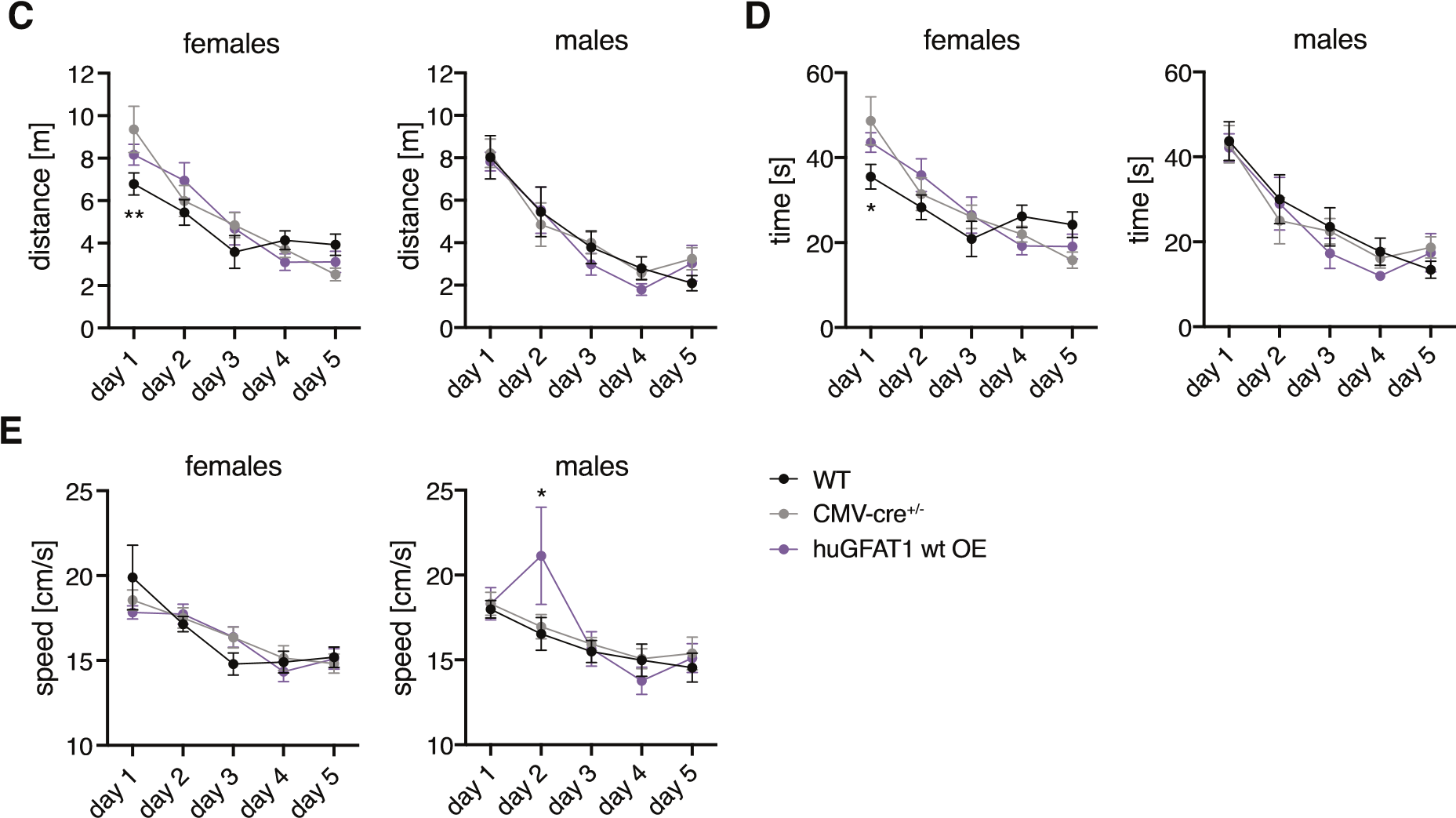


**Figure S5: HBP activation by huGFAT1 wt OE does affect behavior or learning of mice.** (A) Speed measured in the open field test of control and huGFAT1 wt OE mice of both sexes at 3, 15 and 21 months of age. (B) Distance measured in the Y maze test of control and huGFAT1 wt OE mice of both sexes at 3, 15 and 21 months of age. (A-B) Data are presented as mean ± SD (n³4). (C) Distance and (D) Time until the mice reached the hidden platform, and (E) Swimming speed of control and huGFAT1 wt OE mice of both sexes at 4 months of age during the Morris water maze training. (C-E) Data are presented as mean ± SEM (n³6). The mean of four trials per day is plotted. (A-E) Two-way ANOVA, Dunnett’s post-test. Statistical significance was calculated compared to CMV-cre^+/-^ mice at each time point; only significant changes

are indicated. *** p<0.001; ** p<0.01; * p<0.05

## 879 Supplementary tables

880 **Table S1: Number of mice used for body weight measurements shown in** 881 **Figure 4e.**

|  | **huGFAT1 wt OE** | | | | | |
| --- | --- | --- | --- | --- | --- | --- |
|  | **females** | | | **males** | | |
| **months** | **WT** | **Cre+/-** | **huGFAT1 wt OE** | **WT** | **Cre+/-** | **huGFAT1 wt OE** |
| **12** | 15 | 21 | 12 | 12 | 12 | 13 |
| **15** | 14 | 20 | 11 | 12 | 12 | 13 |
| **18** | 14 | 19 | 13 | 12 | 12 | 15 |
| **21** | 13 | 18 | 13 | 12 | 11 | 17 |
| **24** | 11 | 15 | 11 | 11 | 10 | 13 |
| **27** | 7 | 12 | 4 | 9 | 7 | 9 |

882

883 **Table S2: Number of mice used for body weight measurements shown in** 884 **Figure S3d.**

|  | **huGFAT1 G451E OE** | | | | | |
| --- | --- | --- | --- | --- | --- | --- |
|  | **females** | | | **males** | | |
| **months** | **WT** | **Cre+/-** | **huGFAT1 G451E OE** | **WT** | **Cre+/-** | **huGFAT1 G451E OE** |
| **12** | 15 | 21 | 12 | 11 | 12 | 12 |
| **15** | 14 | 20 | 12 | 11 | 12 | 11 |
| **18** | 14 | 19 | 12 | 11 | 12 | 11 |
| **21** | 13 | 18 | 14 | 10 | 11 | 10 |
| **24** | 11 | 15 | 9 | 9 | 10 | 9 |
| **27** | 7 | 12 | 6 | 8 | 8 | 7 |

885
